# Supplementary figures and images for: Identification and biological characterization of pathogen causing sooty blotch of Ardisia crispa (Thunb.) A.DC
Source: PeerJ. 2025 Mar 24;13:e19130. doi: 10.7717/peerj.19130 (PMC11949116; doi:10.7717/peerj.19130)

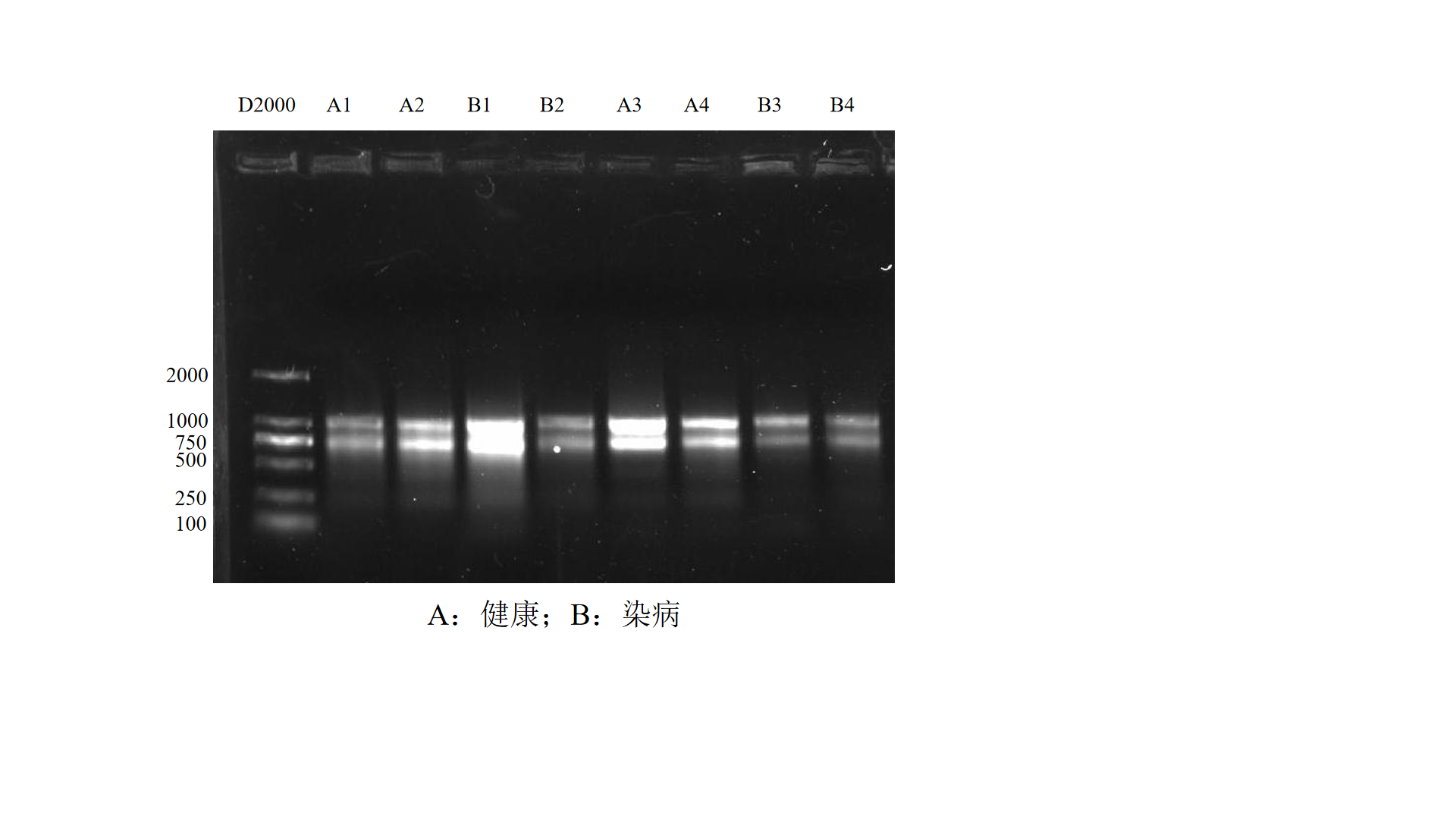
 **
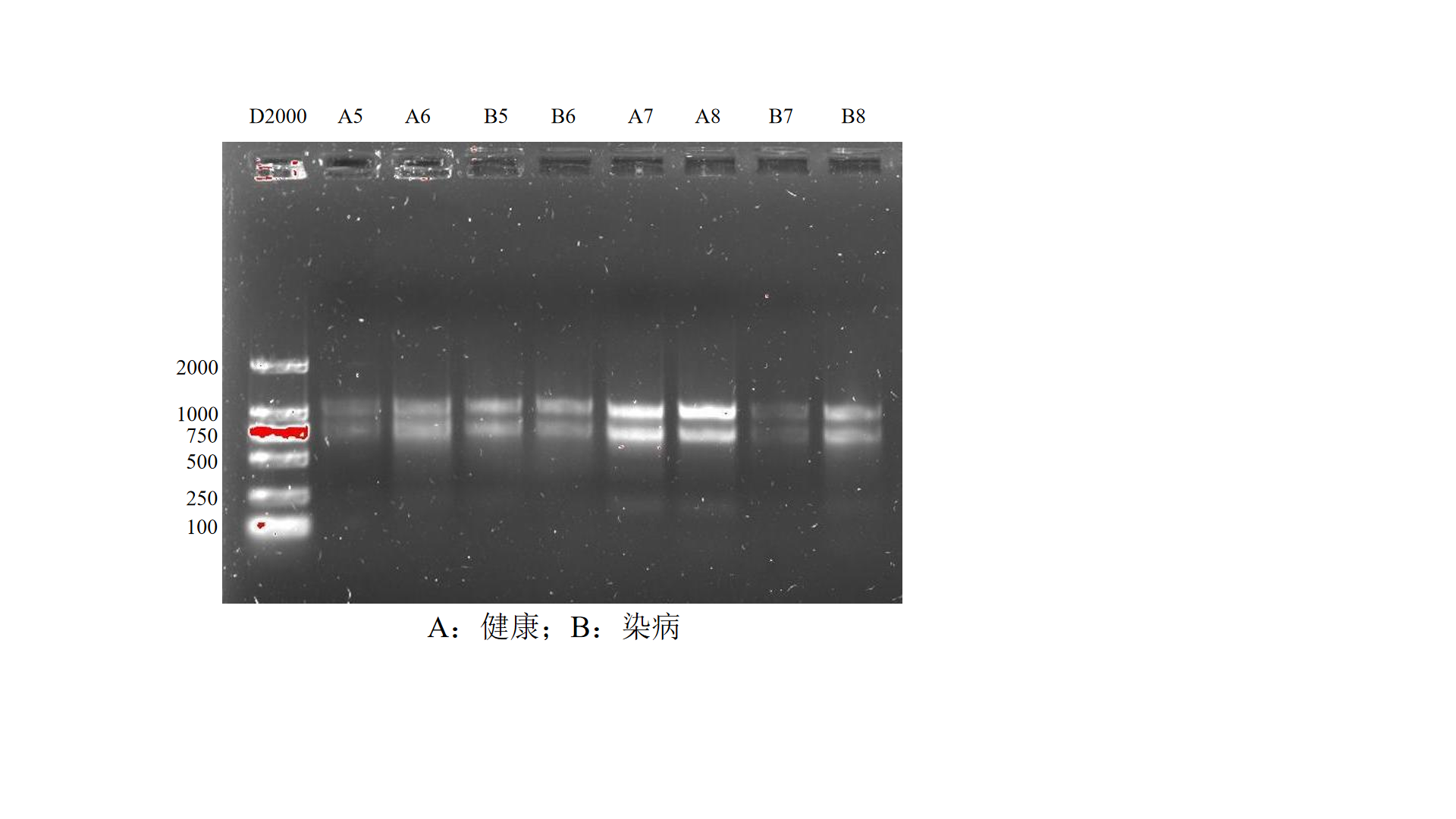
**

Figure S1 the result of RNA extraction. A:healthy leaves; B:disease leaves.

Supplement: Supplemental Information 1 [file peerj-13-19130-s001.docx]

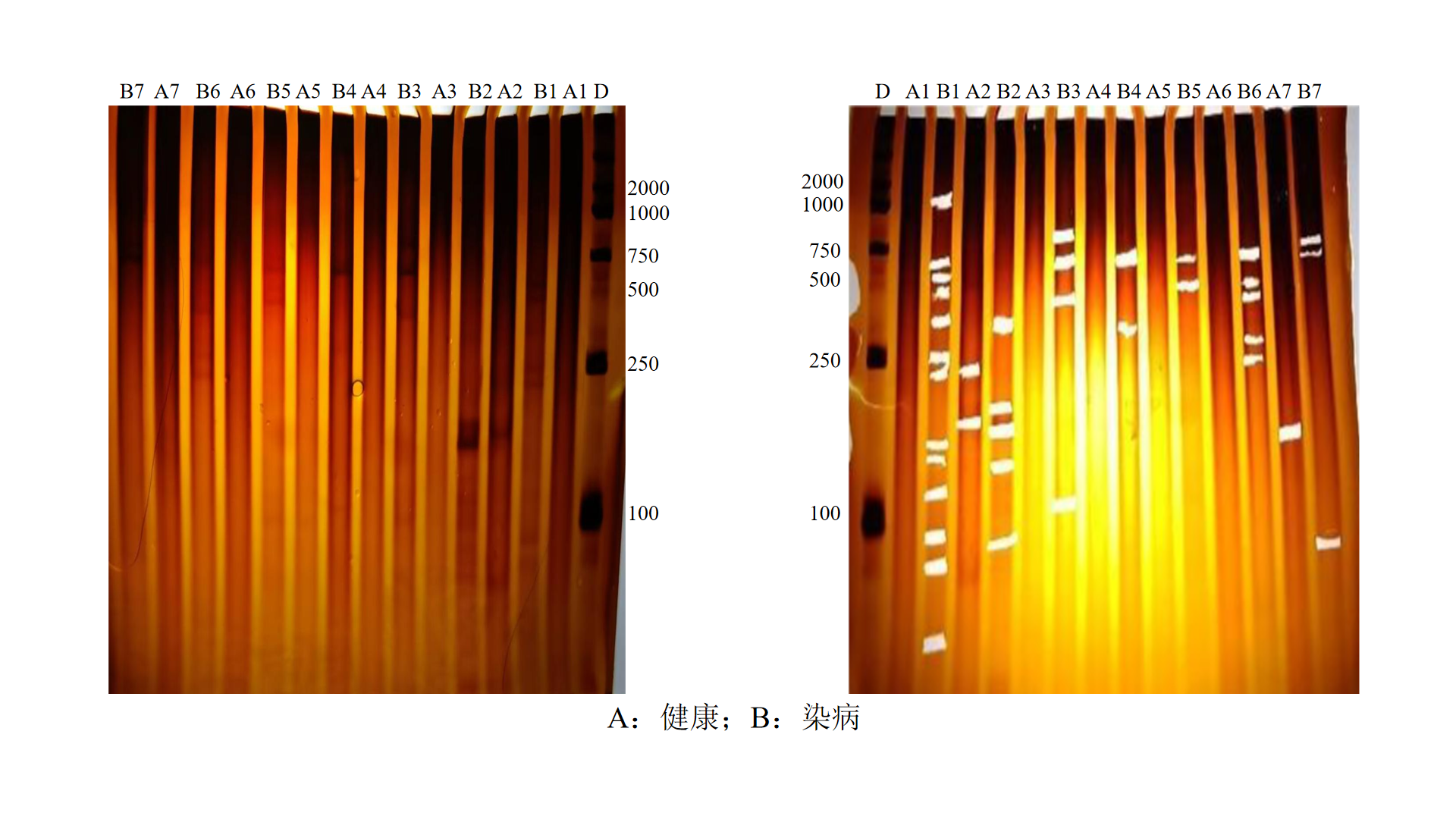


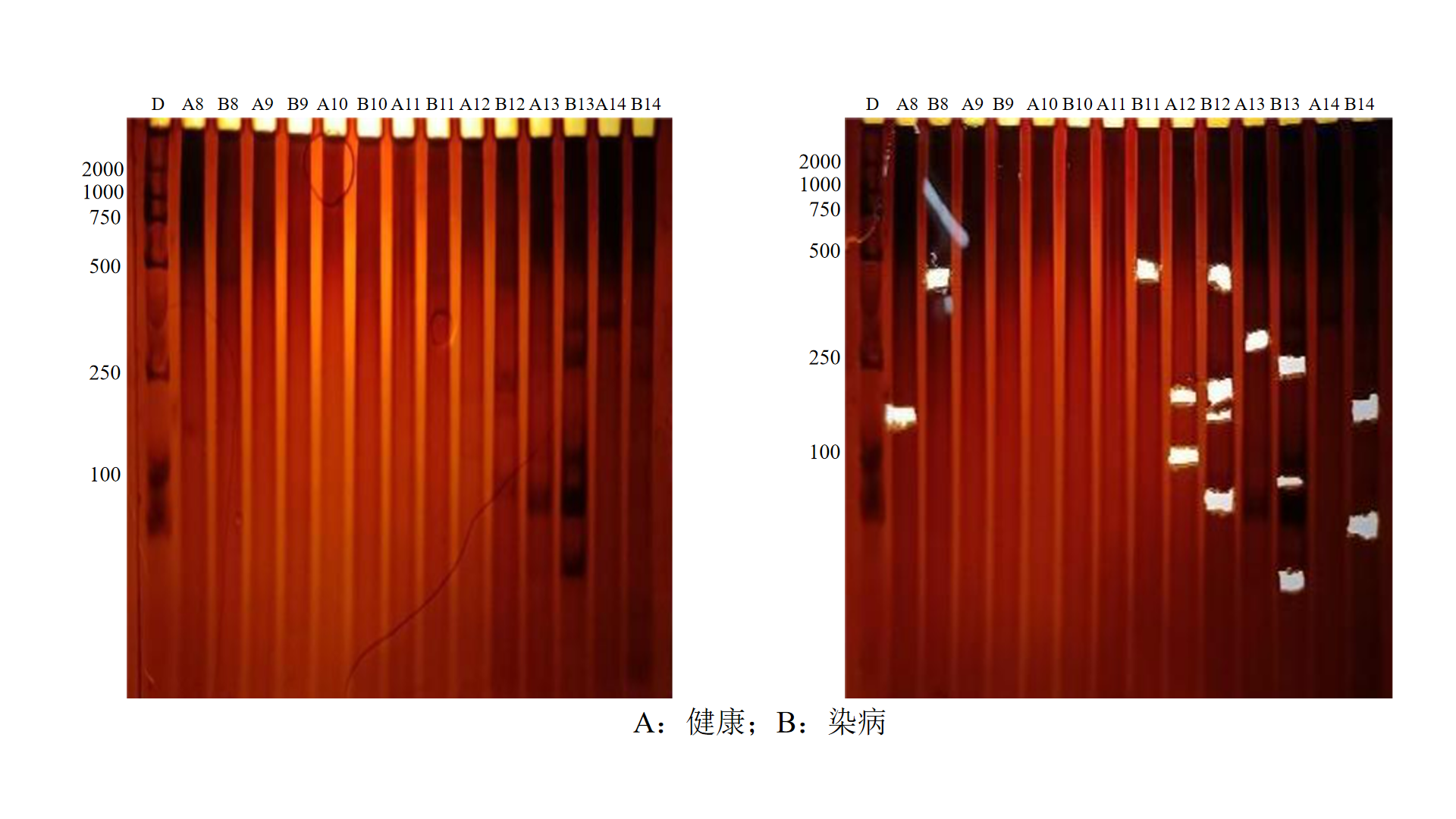


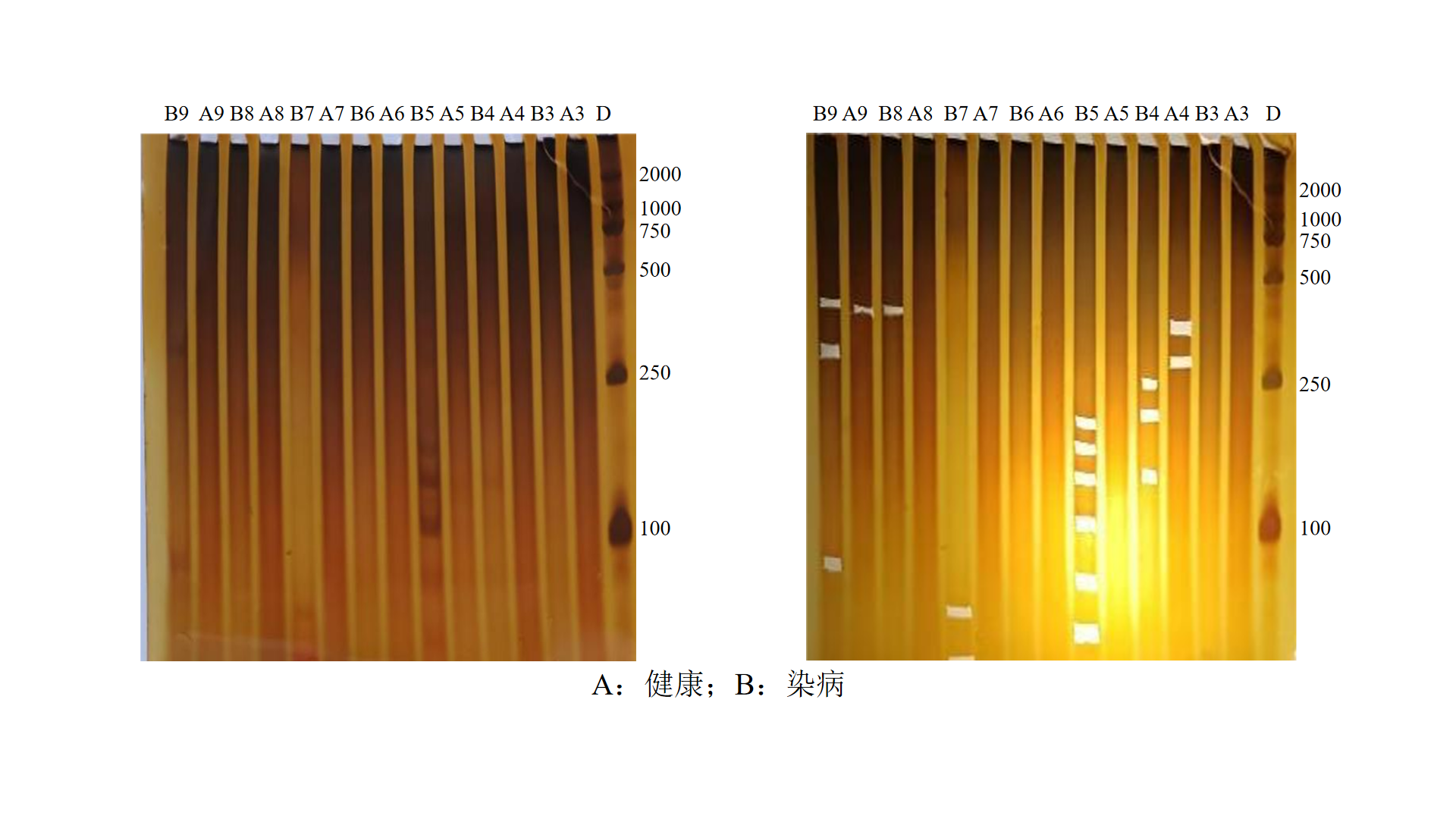


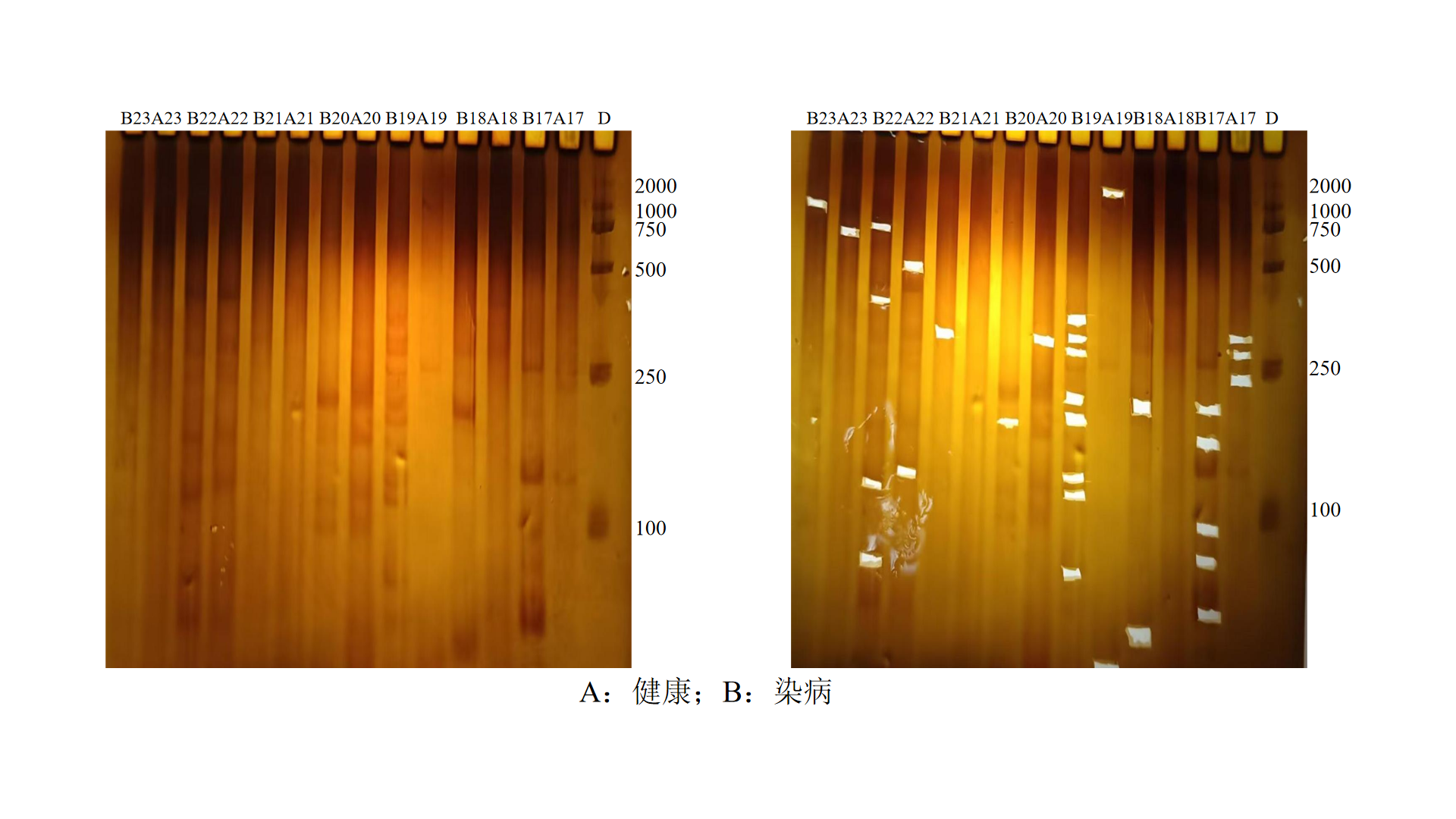


**
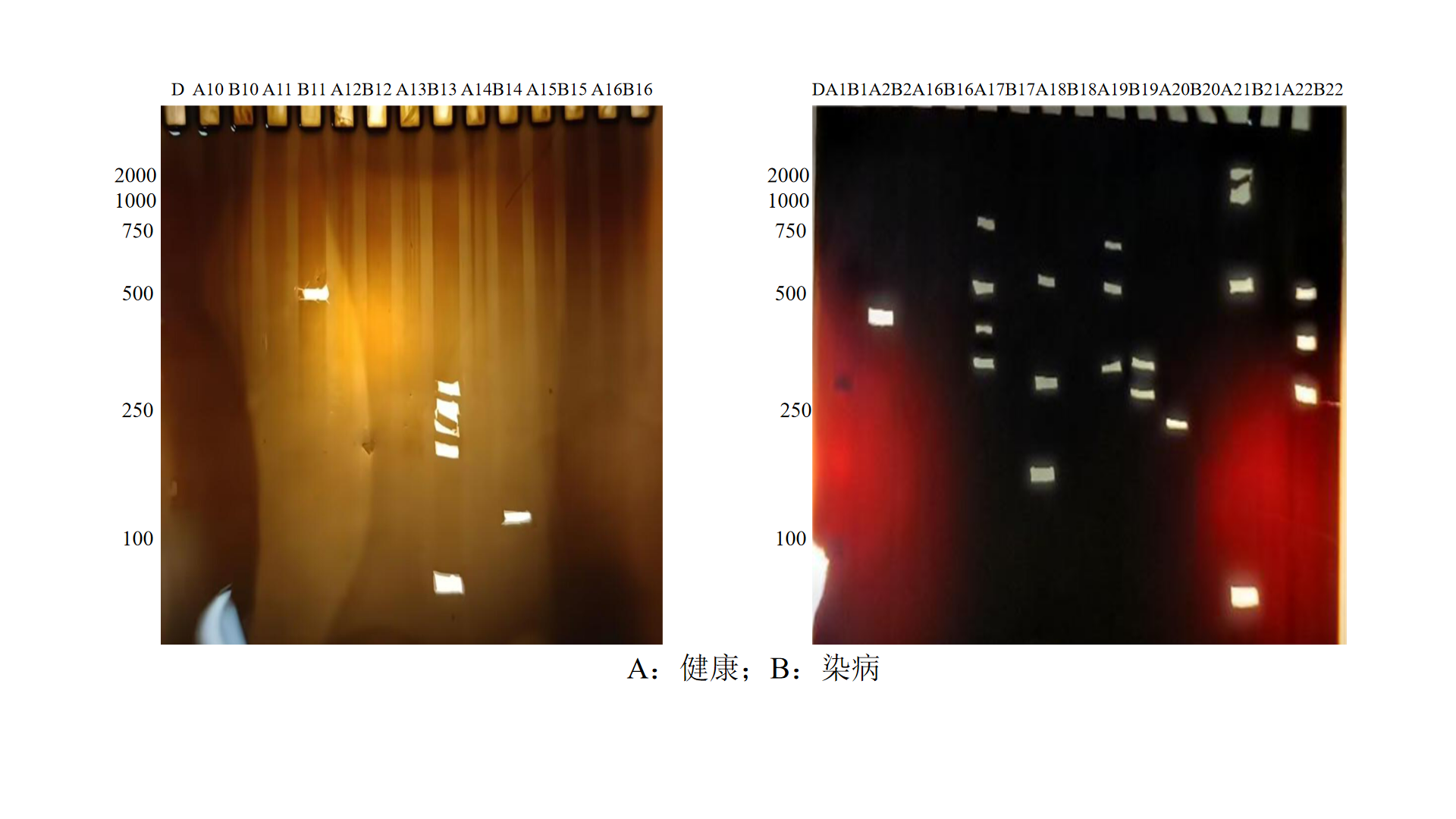
**

**
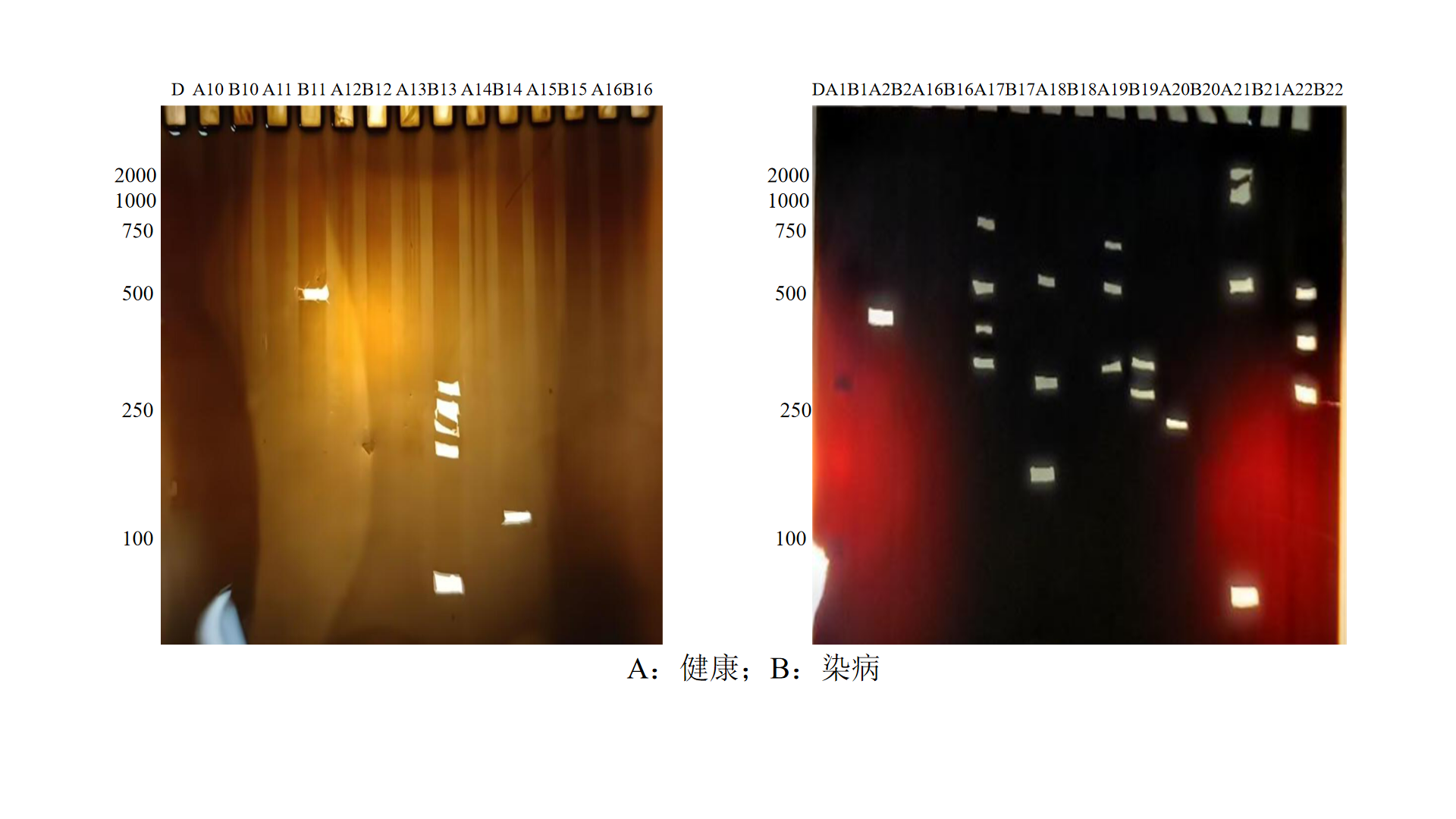
**

Figure S2 Polyacrylamide gel electrophoresis.A:healthy leaves; B:disease leaves.

Supplement: Supplemental Information 2 [file peerj-13-19130-s002.docx]

**
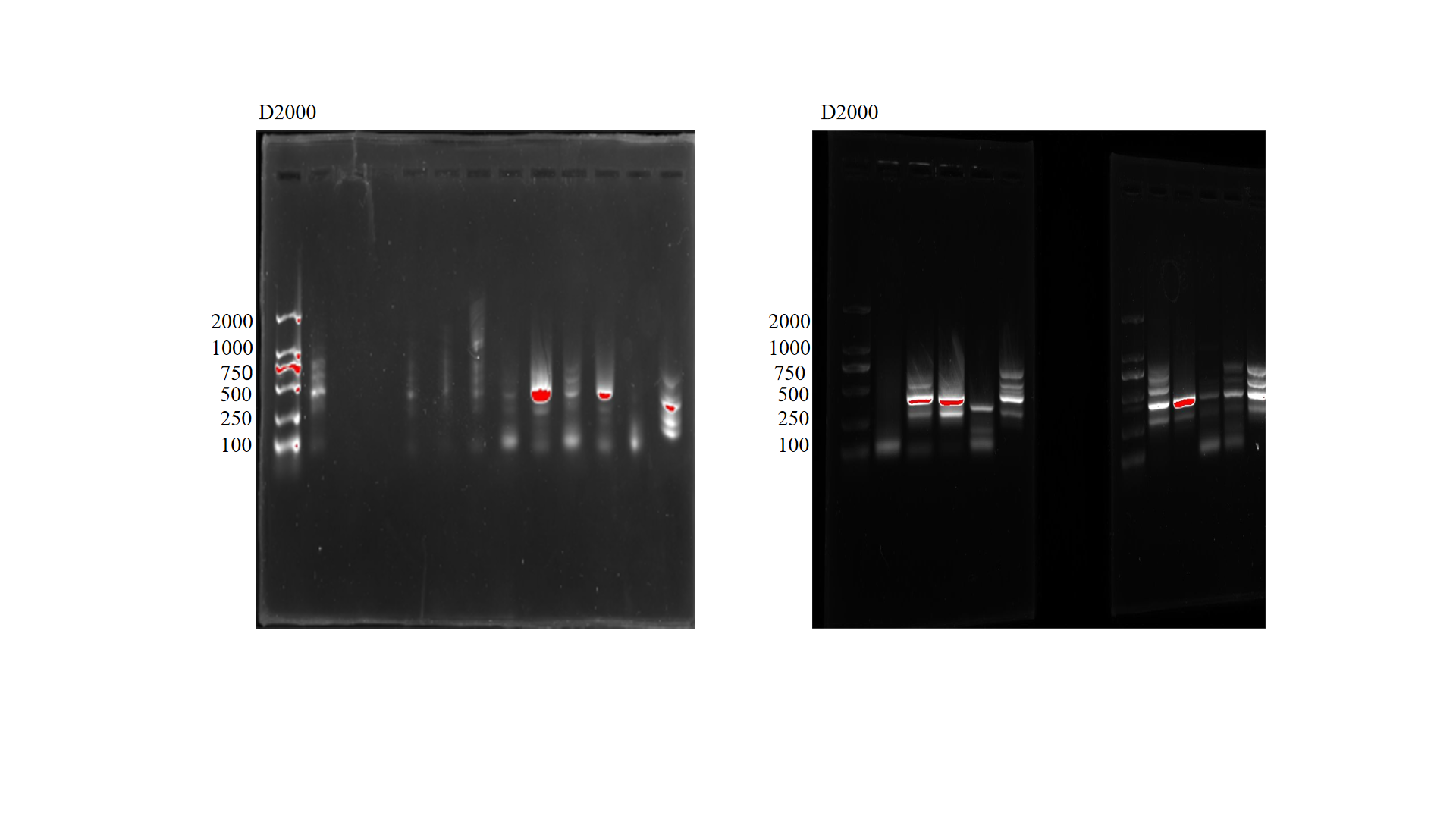

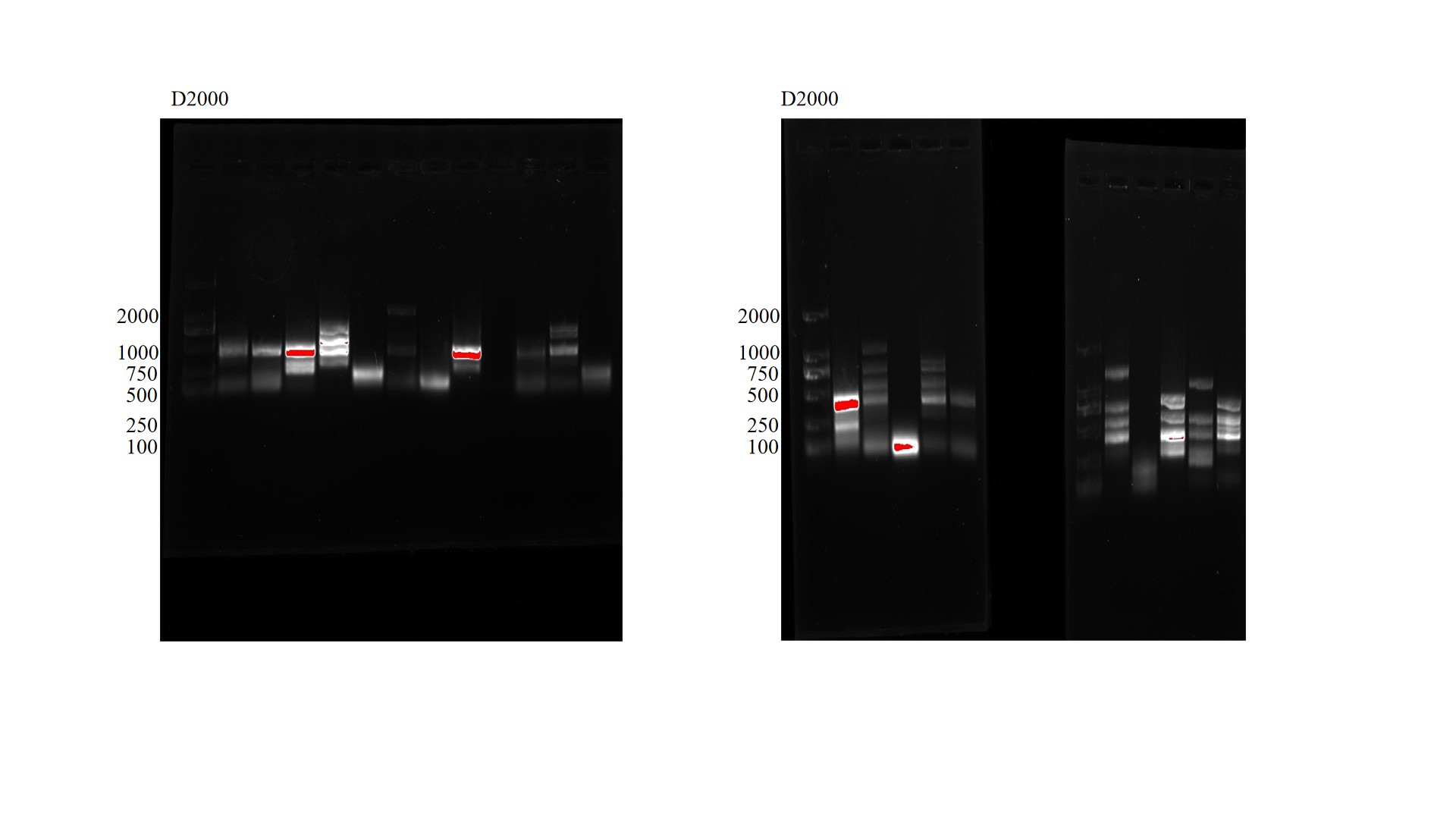
**

**
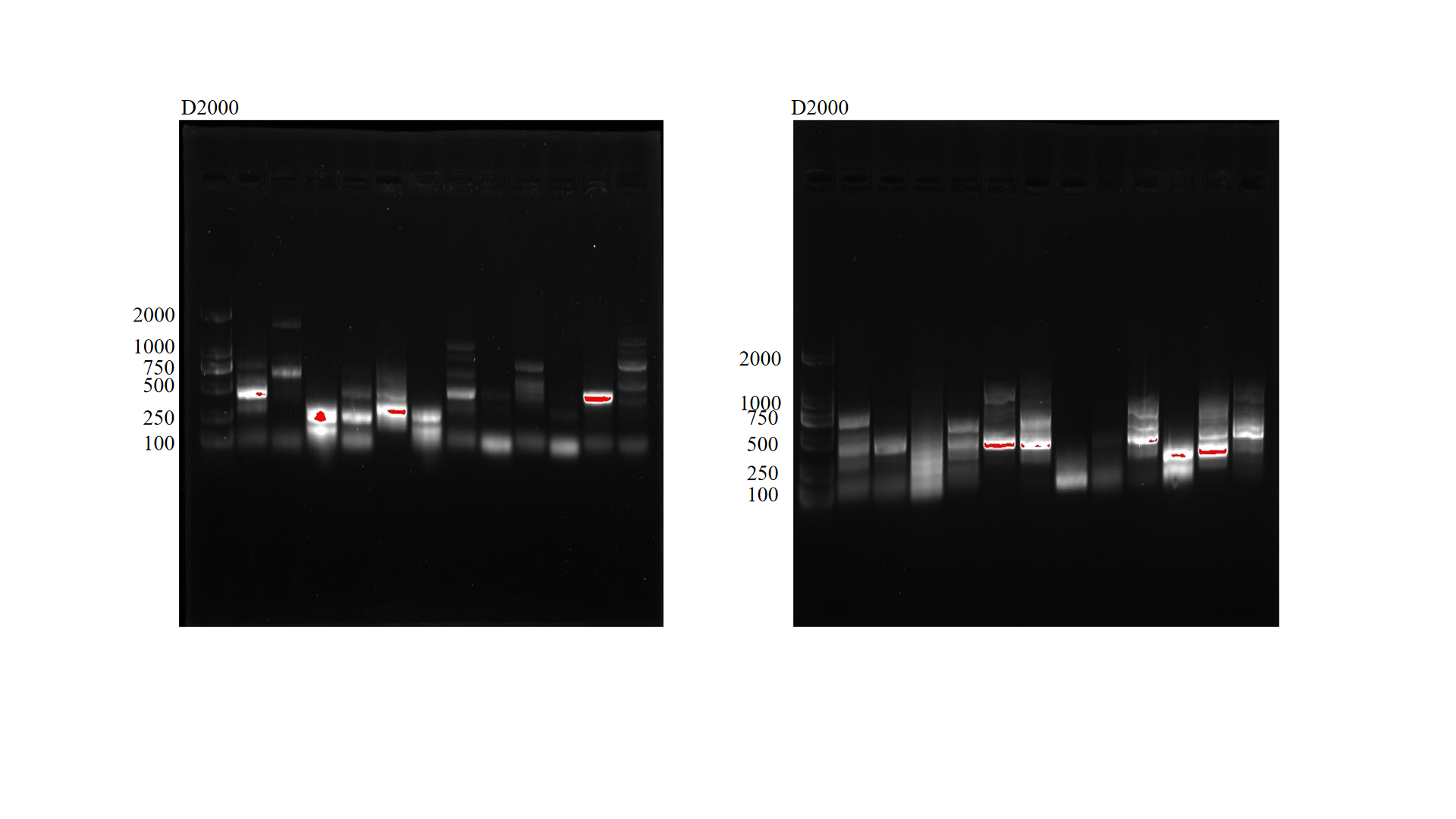
**

**
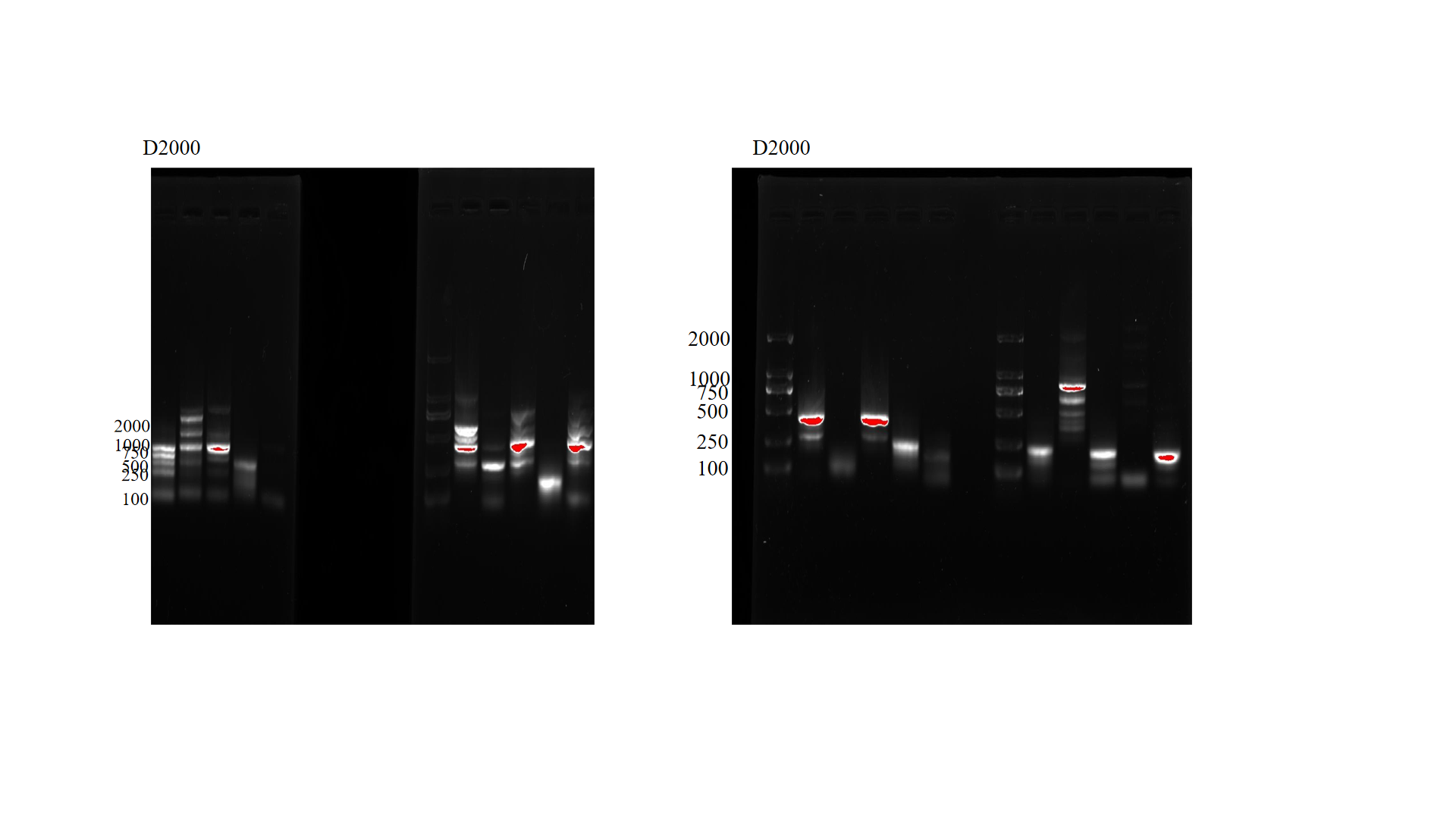
**

**
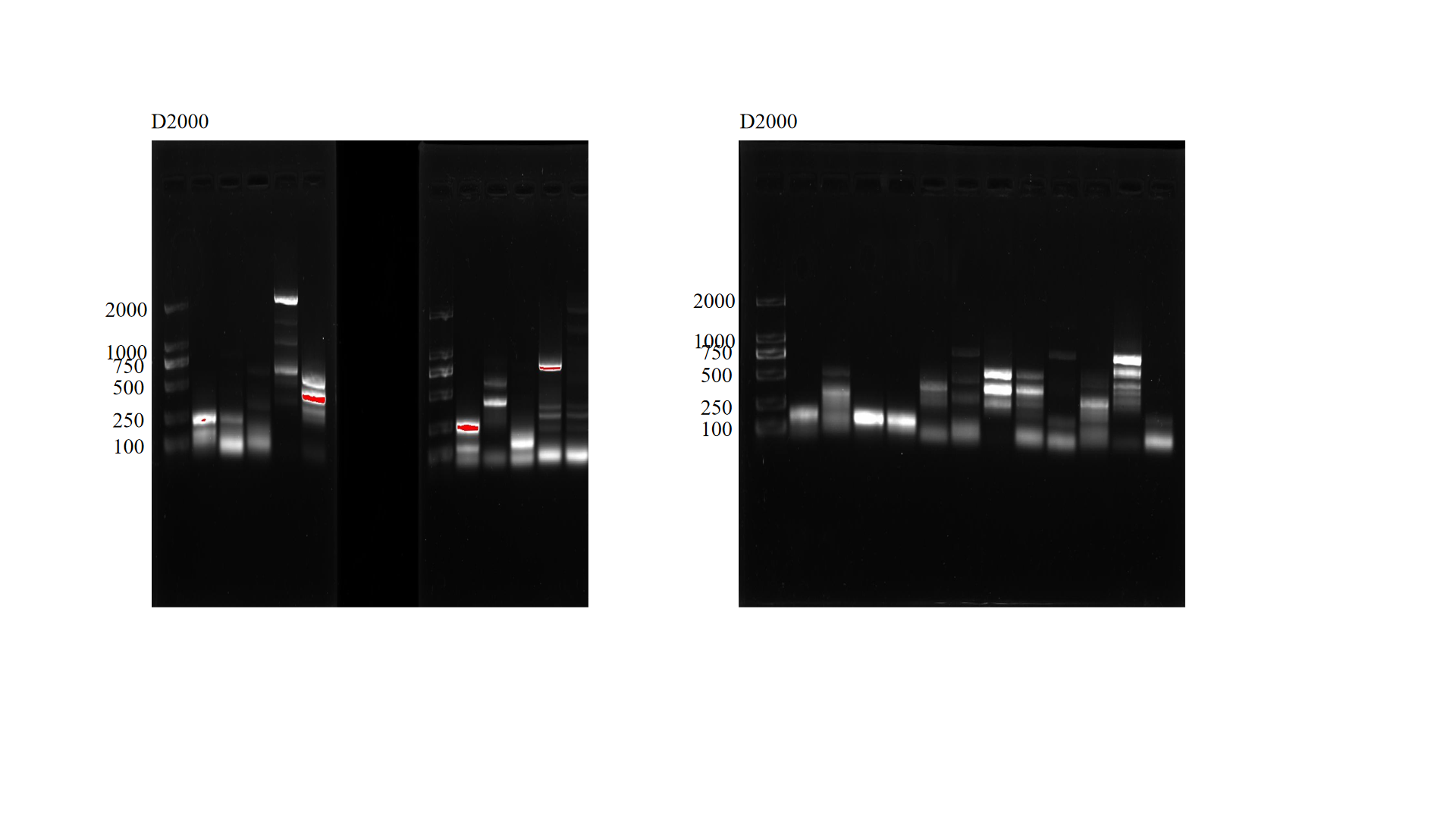
**

**
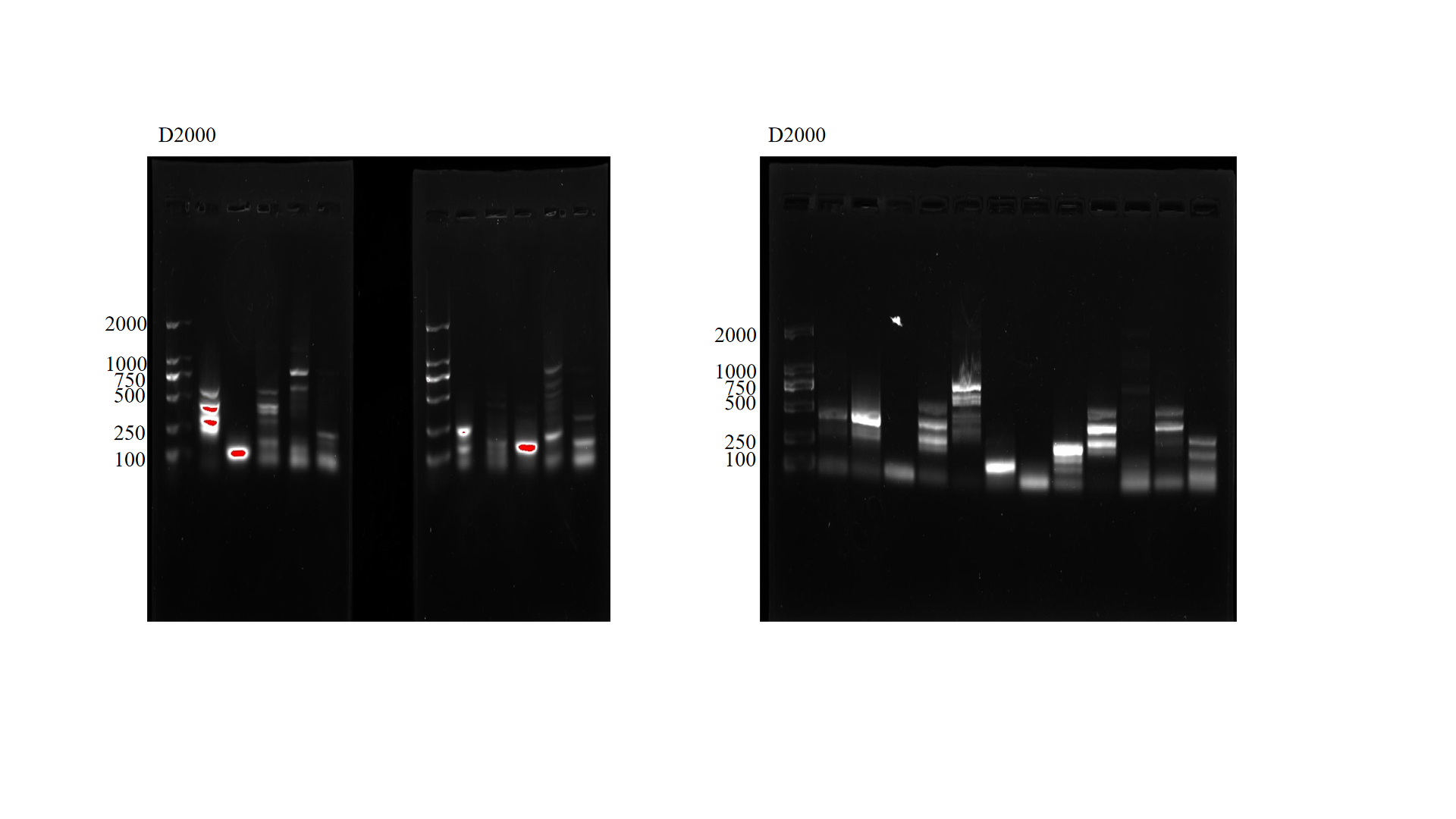
**

**
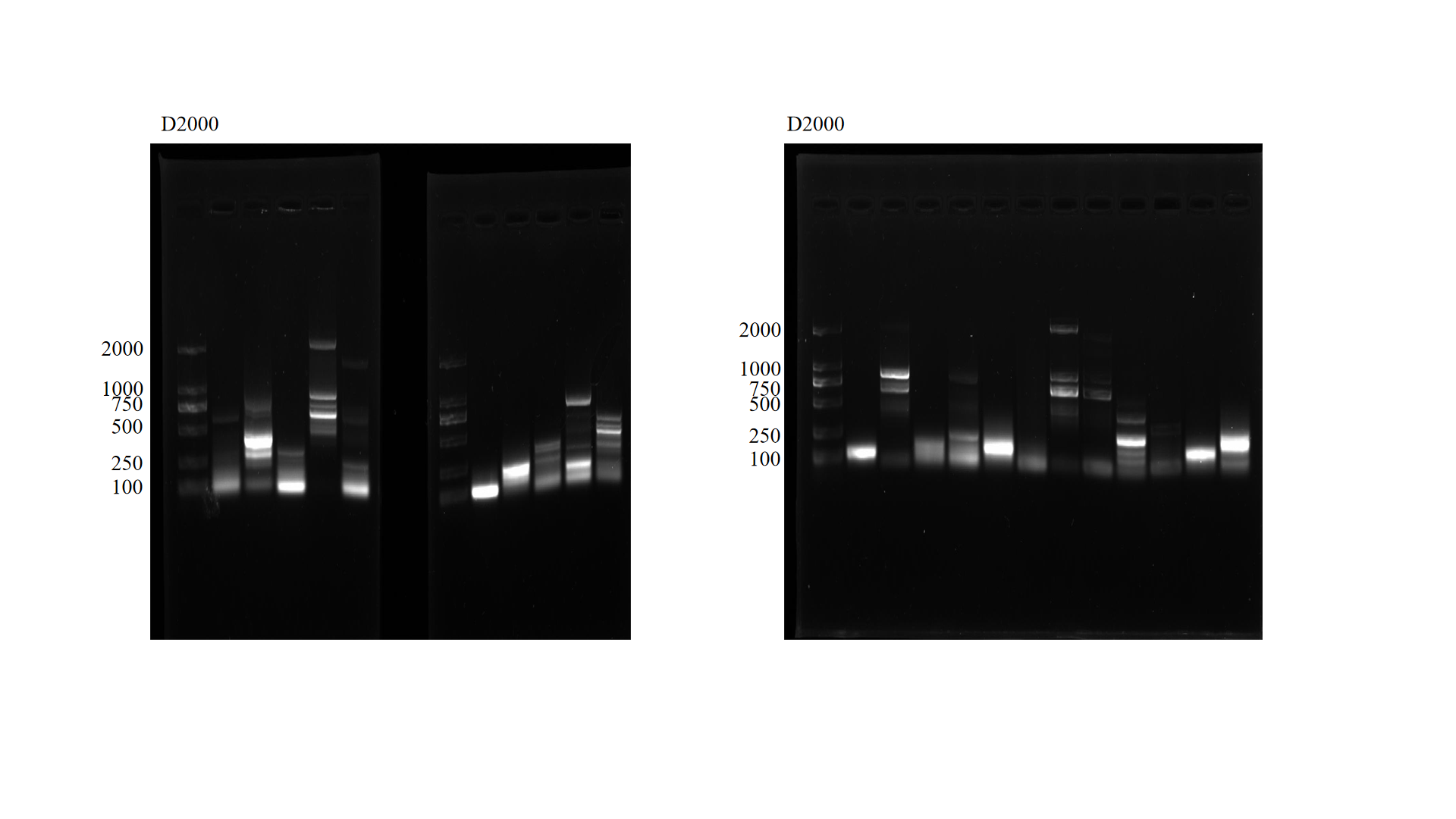
**

**
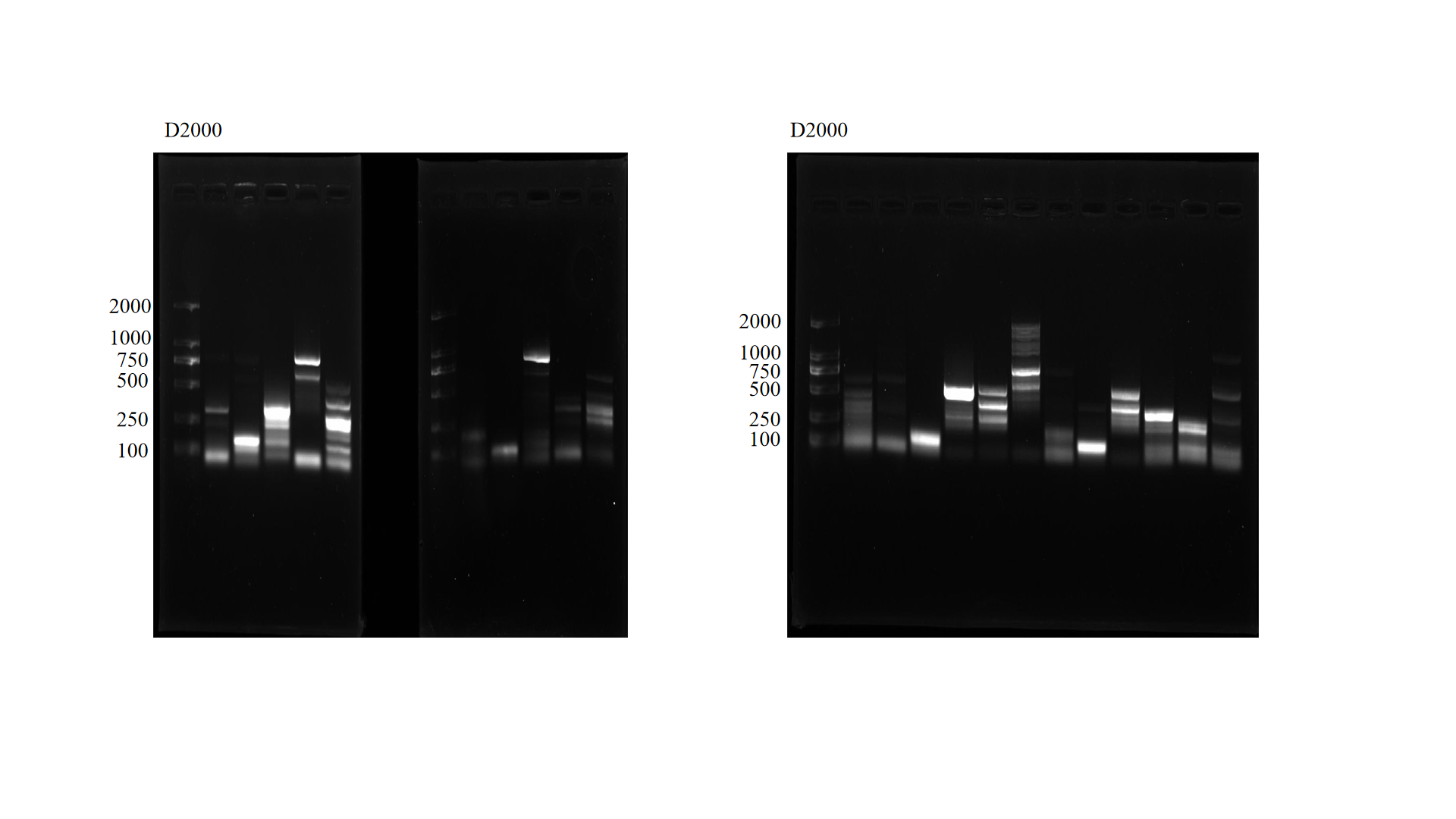
**

**
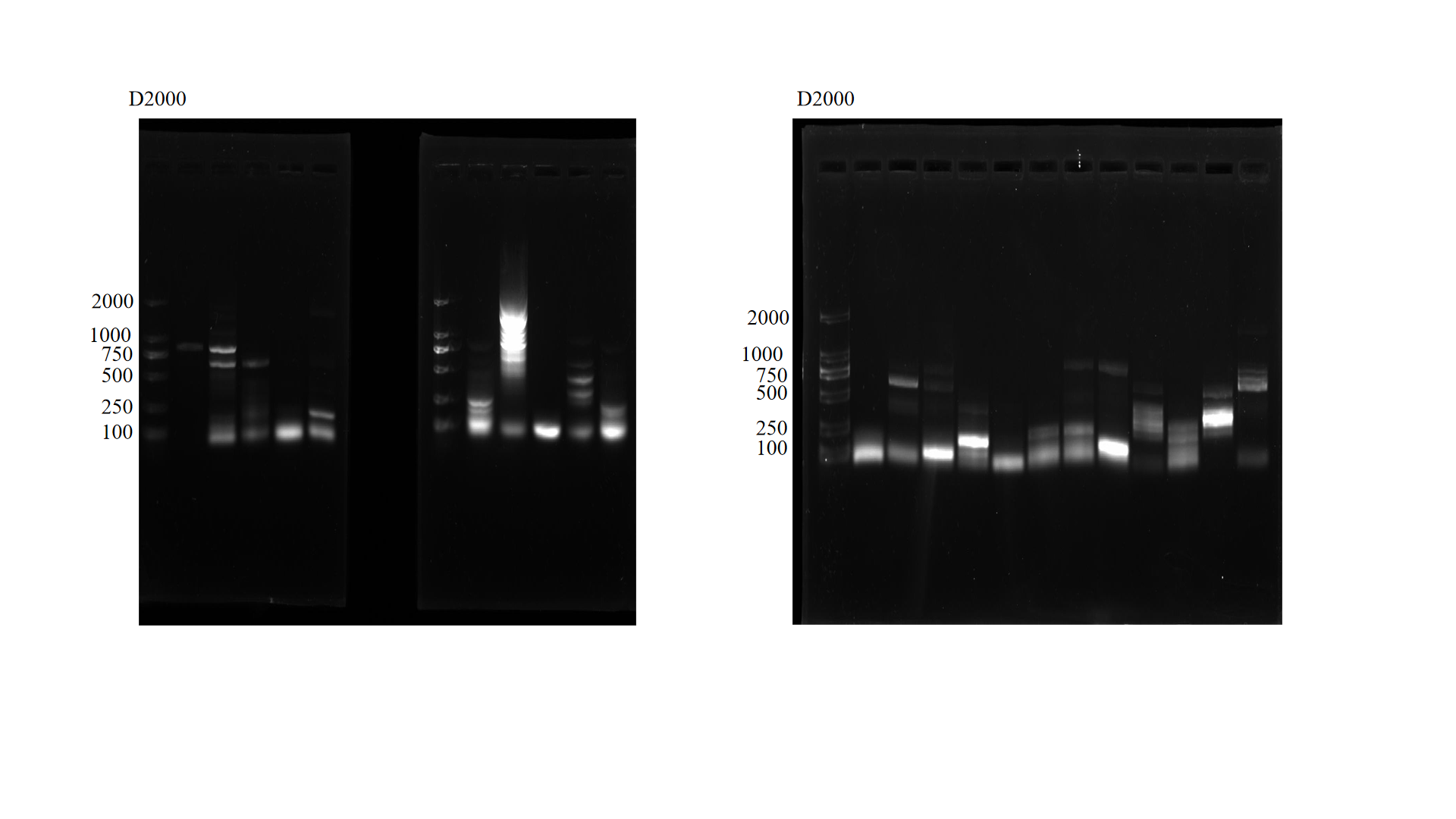
**

Figure S3 DNA recovery from different bands by agarose gel electrophoresis.

Supplement: Supplemental Information 3 [file peerj-13-19130-s003.docx]

**
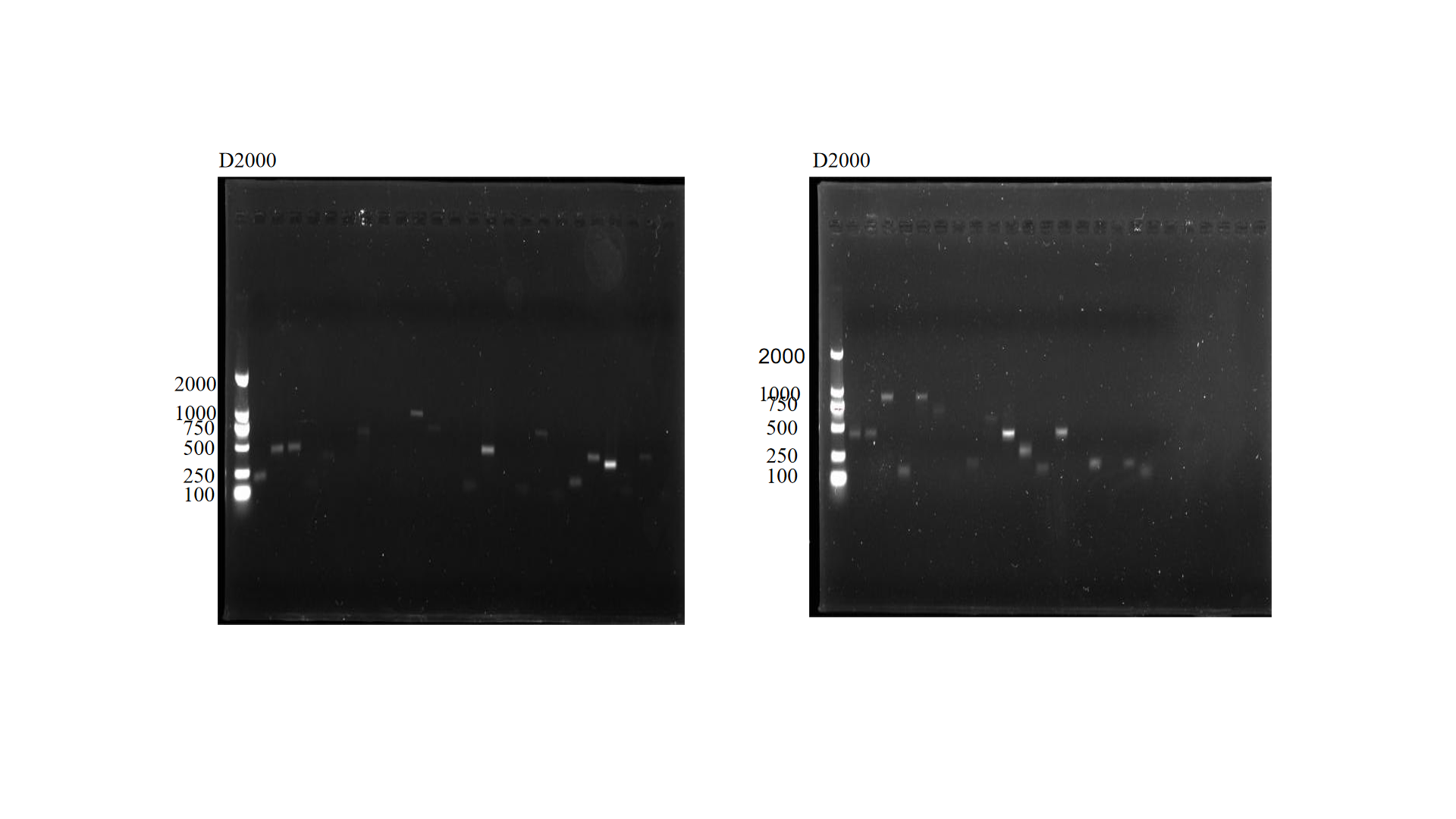
**

**
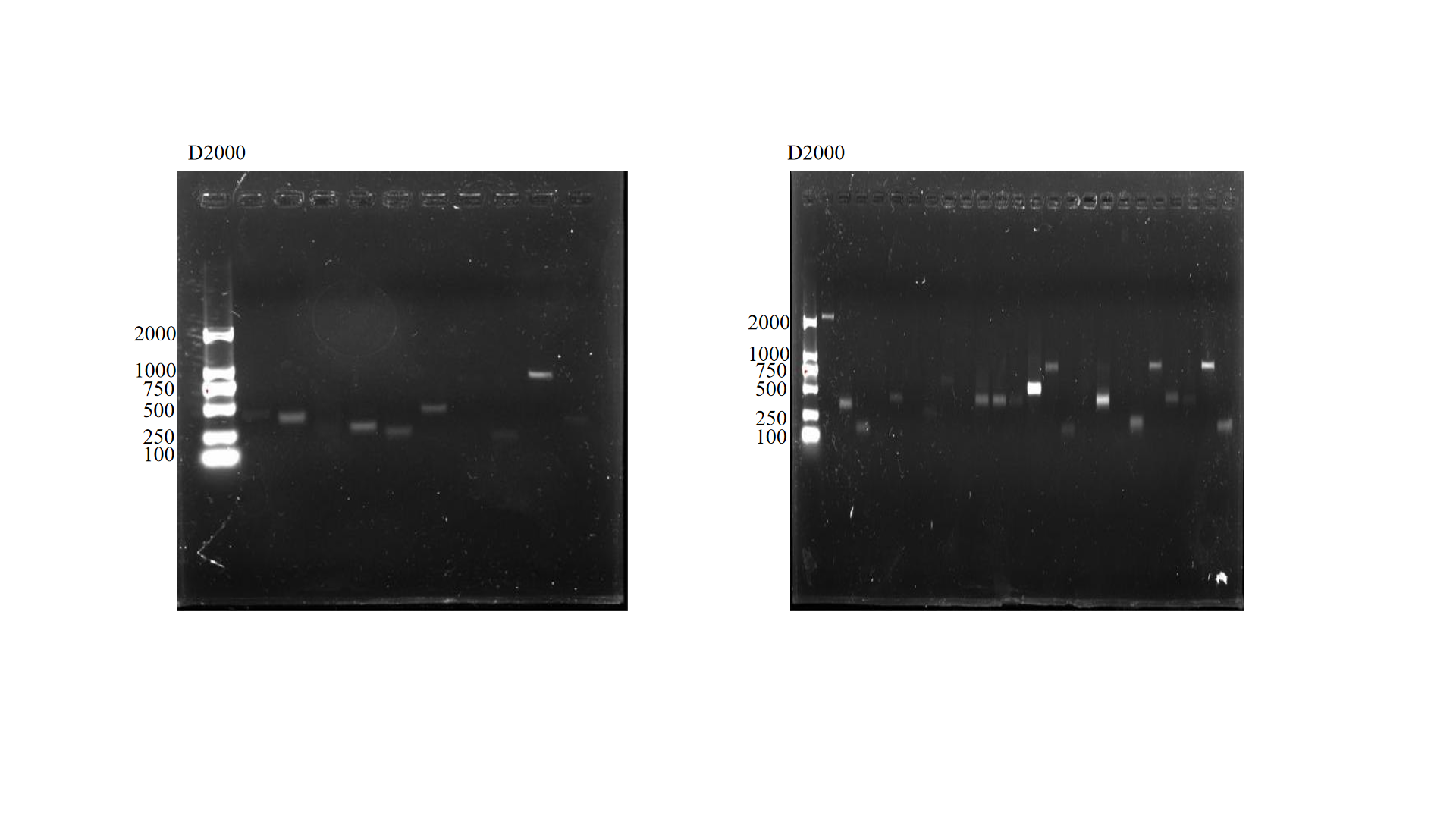
**

**
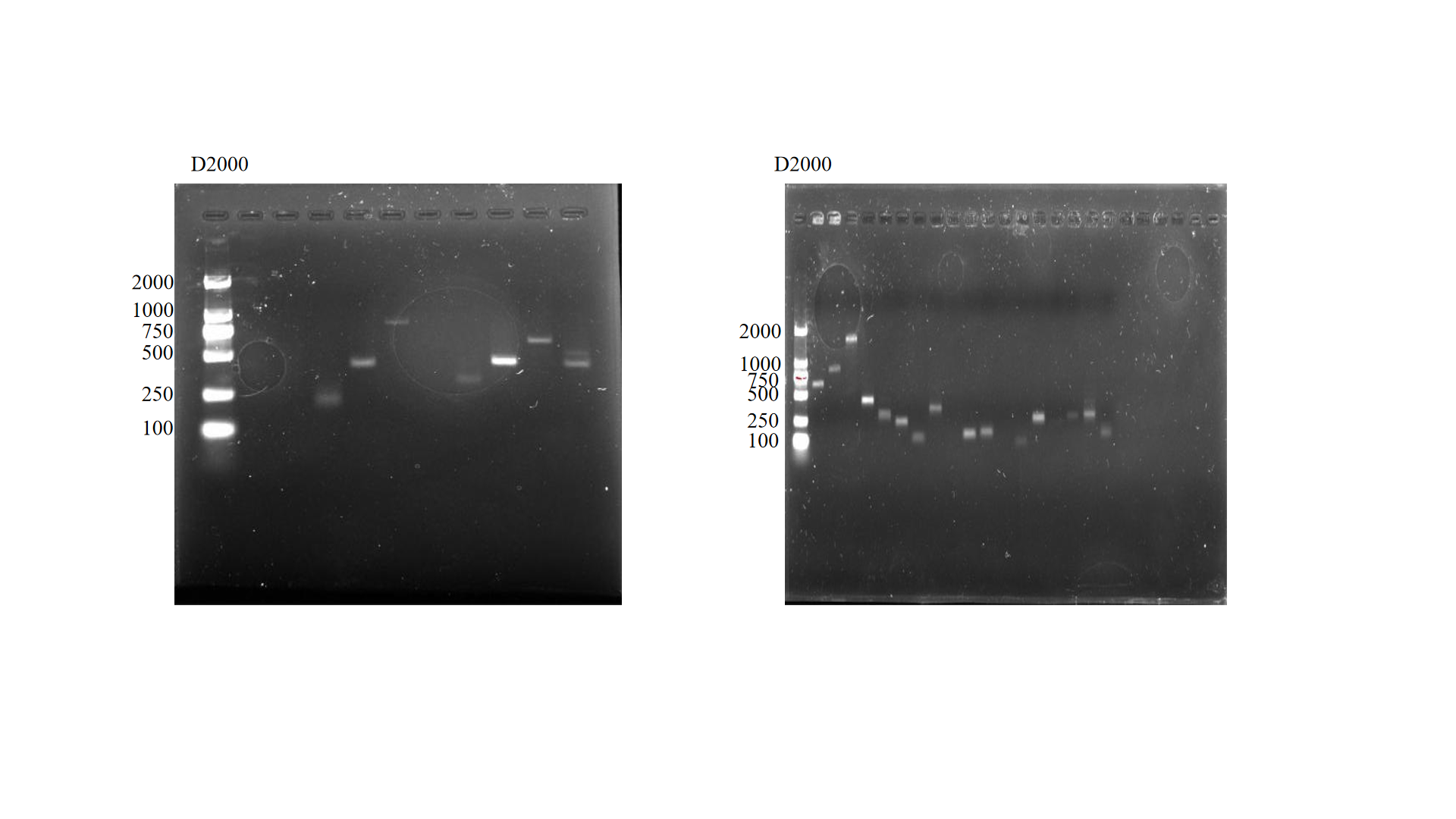
**

Figure S4 Single bands.

Supplement: Supplemental Information 4 [file peerj-13-19130-s004.docx]
