# Supplementary material for: Identification and biological characterization of pathogen causing sooty blotch of Ardisia crispa (Thunb.) A.DC
Source: PeerJ. 2025 Mar 24;13:e19130. doi: 10.7717/peerj.19130 (PMC11949116; doi:10.7717/peerj.19130)
Supplement: Supplemental Information 5 — The colony status of healthy leaf of Ardisia crispa (Thunb.) A.DC. was infected with pathogenic fungi for 3 days, 8 days and 12 days. [file peerj-13-19130-s005.docx]

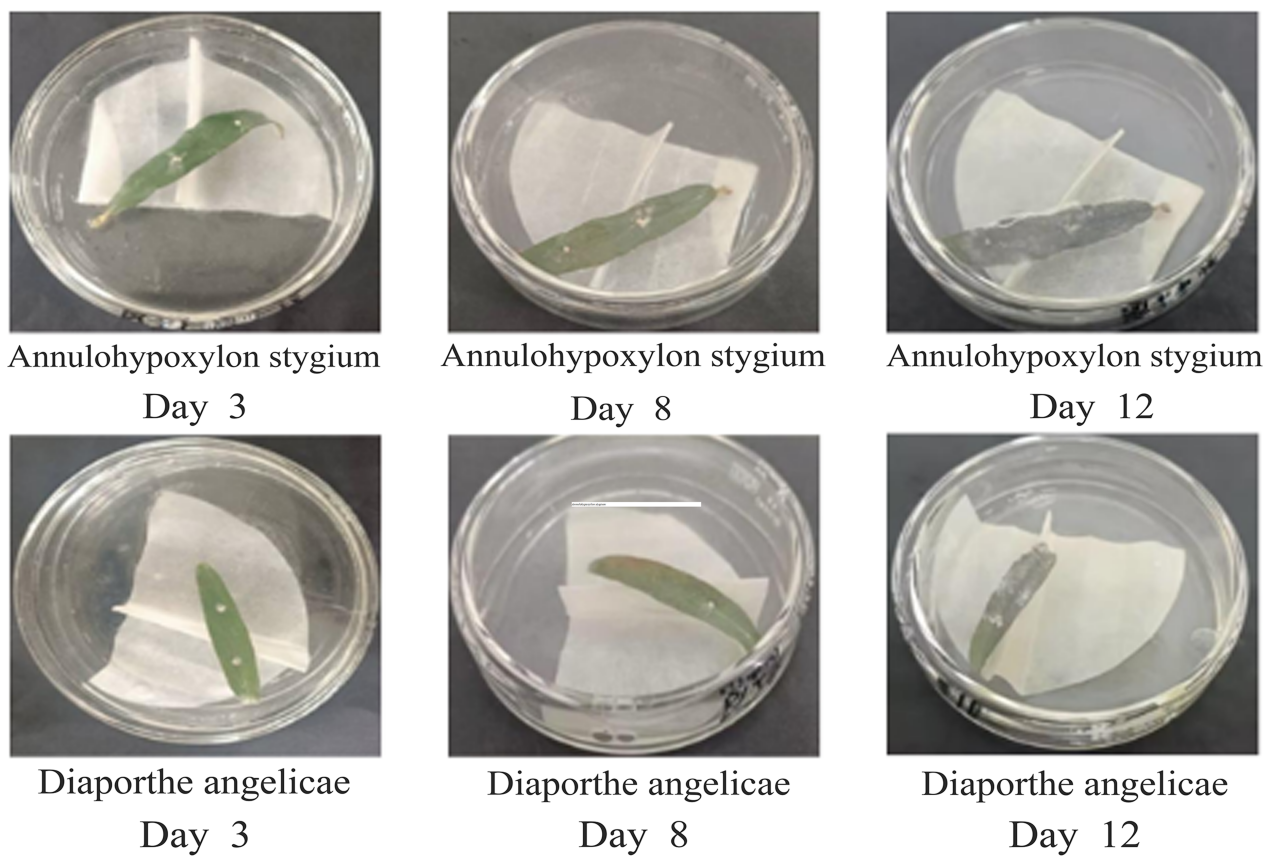


Figure S5 Disease morphology of the two pathogenic fungi in different periods. The colony status of healthy leaf of *Ardisia crispa* (Thunb.) A.DC. was infected with pathogenic fungi for 3 days, 8 days and 12 days.
